# Acute Splenic Sequestration Crisis in a 70-Year-Old Patient With Hemoglobin SC Disease

**DOI:** 10.1177/2324709616638363

**Published:** 2016-03-16

**Authors:** John J. Squiers, Anthony G. Edwards, Alberto Parra, Sandra L. Hofmann

**Affiliations:** 1 University of Texas Southwestern Medical Center, Dallas, TX, USA

**Keywords:** sickle cell disease, hemoglobinopathies, transfusion, HbSC disease

## Abstract

A 70-year-old African American female with a past medical history significant for chronic bilateral shoulder pain and reported sickle cell trait presented with acute-onset bilateral thoracolumbar pain radiating to her left arm. Two days after admission, Hematology was consulted for severely worsening microcytic anemia and thrombocytopenia. Examination of the patient’s peripheral blood smear from admission revealed no cell sickling, spherocytes, or schistocytes. Some targeting was noted. A Coombs test was negative. The patient was eventually transferred to the medical intensive care unit in respiratory distress. Hemoglobin electrophoresis confirmed a diagnosis of hemoglobin SC disease. A diagnosis of acute splenic sequestration crisis complicated by acute chest syndrome was crystallized, and red blood cell exchange transfusion was performed. Further research is necessary to fully elucidate the pathophysiology behind acute splenic sequestration crisis, and the role of splenectomy to treat hemoglobin SC disease patients should be better defined.

A 70-year-old African American female with a past medical history significant for hypertension, type 2 diabetes mellitus, chronic bilateral shoulder pain, and self-reported sickle cell trait presented with acute-onset bilateral thoracolumbar pain radiating to her left arm. She described her back pain as a “constant tearing” but denied chest pain, dyspnea, abdominal pain, and dysuria. Other than pain elicited by palpation of the bilateral lumbar area and limited range of motion in the left shoulder, the patient’s physical examination was unremarkable, with normal cardiac and respiratory exams and no appreciable hepatosplenomegaly. Computed tomography (CT) angiogram performed in the emergency department was negative for aortic dissection. Vaso-occlusive crisis of sickle cell disease was briefly considered, but the patient’s insistence that she had only sickle cell trait and an absence of prior sickle cell crises in her history eliminated this diagnosis from the differential. She was admitted to the hospitalist service for intractable back pain and placed on patient-controlled analgesia. Laboratory values on admission were hemoglobin (Hb) 8.7 g/dL (usual baseline Hb = 9 g/dL), hematocrit 28%, white cell count (WBC) 8.8 × 10^9^/L, platelets 284 × 10^9^/L, and mean corpuscular volume 71 fL.

Two days after admission, Hematology was consulted for severely worsened microcytic anemia and thrombocytopenia. Laboratory values were now Hb 4.4 g/dL, WBC 21.6 × 10^9^/L, and platelets 89 × 10^9^/L. On examination, the patient had developed acute-onset dyspnea with tachypnea of 30 to 40 breaths/minute, increased work of breathing, and oxygen desaturations to 86% on room air. Her temperature was 38.1°C. Inflammatory markers were elevated: erythrocyte sedimentation rate 57 mm/h, C-reactive protein 22.3 mg/dL, lactate dehydrogenase 1164 U/L, creatinine kinase 1647 U/L, fibrinogen 501 mg/dL. Serum haptoglobin 181 mg/dL and serum total bilirubin 0.6 mg/dL were within normal limits. Electrocardiogram showed no changes from prior examinations. Examination of the patient’s peripheral blood smear from admission revealed no cell sickling, spherocytes, or schistocytes. Some targeting was noted. A Coombs test was negative.

The patient’s tachypnea, temperature, leukocytosis, and increased inflammatory markers were consistent with systemic inflammatory response syndrome, most likely in the setting of sepsis, though no infectious source was immediately apparent. The patient was transferred to the medical intensive care unit for respiratory distress. Given that the patient’s peripheral blood smear lacked any evidence of active hemolysis, the patient’s acute anemia was felt to be more likely due to acute blood loss. However, there was no clinical evidence for an external or a gastrointestinal tract bleed. The initial CT angiogram was reexamined for a retroperitoneal bleed that may have also been the cause of the patient’s back pain on presentation, but no evidence for a bleed was discovered. Her symptoms were unresponsive to intravenous morphine, and the patient was intubated. Empiric antibiotics were started for presumed sepsis after chest radiography demonstrated bibasilar atelectasis concerning for pneumonia. Overnight, she was transfused with 3 units of packed red blood cells.

The next morning, laboratory values were Hb 8.1 g/dL, WBC 14.8 × 10^9^/L, and platelets 46 × 10^9^/L. Spherocytes were identified on a peripheral smear, but Coombs test remained negative. The patient’s worsening thrombocytopenia was felt to be secondary to sepsis. The spherocytes present on peripheral smear raised concern for a hemolytic process, and because no source of acute blood loss had been identified, a workup was initiated for Coombs-negative hemolytic anemia. The differential for this phenomenon includes certain infections, hypersplenism, and cold agglutinin hemolysis, among other less common etiologies. Given the patient’s history, clinical concern for sepsis, and peripheral smear findings, we felt that infection was the most likely explanation for her hemolysis.

As part of the work-up in this perplexing case of acute anemia, Hb electrophoresis was also ordered to confirm the patient’s diagnosis of sickle cell trait. Instead, Hb electrophoresis demonstrated that HbS and HbC each comprised greater than 40% of total Hb, revealing underlying hemoglobin SC (HbSC) disease rather than sickle cell trait. With this information, a diagnosis of acute splenic sequestration crisis (ASSC) complicated by acute chest syndrome (ACS) was crystallized, indicating RBC exchange transfusion for therapeutic intervention. A Quinton catheter was placed, and the patient underwent exchange with 5 units of packed red blood cells, after which the patient reported complete resolution of her back pain. Laboratory values were Hb 10.3 g/dL, WBC 11.4 × 10^9^/L, and platelets 39 × 10^9^/L. The patient was extubated shortly thereafter without recurrence of dyspnea or tachypnea.

The patient’s hospital course following RBC exchange was relatively uncomplicated. She remained tachycardic with low-grade fevers for several days while she was treated with antibiotics for pneumonia. CT abdomen after the RBC exchange revealed an enlarged, heterogeneous spleen with multifocal areas of low density, consistent with ASSC. Further findings included irregular splenic contour and scattered calcifications within splenic parenchyma most likely related to prior infarcts (Figure 1). After 5 days, the patient was transferred out of the medical intensive care unit, and she was discharged home 2 days later. Immediately prior to discharge, laboratory values were Hb 7.8 g/dL, WBC 8.0 × 10^9^/L, and platelets 133 × 10^9^/L. Approximately 2 months following discharge, the patient returned to her primary care physician for follow-up. She was without any physical complaints, and her laboratory values were at her baseline: Hb 9.1 g/dL, WBC 11.4 × 10^9^/L, and platelets 207 × 10^9^/L.

**Figure 1.**
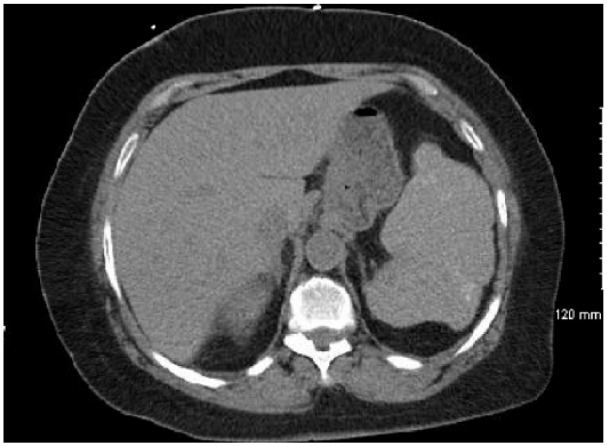
CT abdomen following red blood cell exchange transfusion demonstrated enlarged spleen consistent with acute splenic sequestration crisis.

This case illustrates ASSC complicated by ACS in an elderly woman presenting with isolated back pain and undiagnosed HbSC disease. HbSC disease is commonly thought of as a variant of sickle cell disease. The condition arises from compound heterozygous mutations in the globin chain—1 gene for “S” hemoglobin (Glu-Val substitution at position 6 of the β-globin chain) and 1 gene for “C” hemoglobin (Glu-Lys substitution at the same position). In HbSC, patients’ RBCs are composed of equal levels of HbS and HbC. The combination of these relatively benign traits results in a moderately severe phenotype because HbC enhances the pathogenicity of HbS via the dehydration of SC RBCs.^1^

The mechanism by which HbC dehydrates RBCs is thought to be the volume-dependent upregulation of K-Cl cotransport that produces an increased K^+^ efflux and a concomitant loss of intra-erythorcytic water. The HbC-dependent efflux of water from RBCs results in microcytosis and, due to an increased surface to volume ratio of the dehydrated erythrocytes, targeting on peripheral smear.^1,2^ The findings of microcytosis and targeting, both of which were documented early in the patient’s hospital course, can serve as diagnostic clues in favor of HbSC disease because neither is characteristically present in sickle cell trait, which lacks the HbC necessary to drive erthrocytic dehydration.

Typically, HbSC disease manifests similarly to sickle cell disease but less mildly and less frequently, so HbSC patients have increased life expectancy. Some exceptions to this general rule have been noted: osteonecrosis, especially in the shoulder, occurs with nearly equal frequency (explaining this patient’s chronic bilateral shoulder pain), and ACS may actually have increased mortality in HbSC disease.^1^ ASSC is commonly encountered in children with homozygous sickle cell disease, but adults with sickle cell disease rarely experience ASSC because recurrent episodes of splenic infarction during childhood result in splenic scarring, atrophy, fibrosis, and, ultimately, functional asplenism.^3^ ASSC remains a risk for adult HbSC patients because their splenic function is often preserved.^4^

One of the major questions raised by this case is why the patient’s first manifestation of HbSC disease was ASSC at such a late stage in her life. Although ASSC is a common cause of death among young children with sickle cell disease, reports of HbSC adults manifesting with ASSC are limited to isolated case reports and a single case series comprising 9 patients.^5^ Although ASSC in such cases classically presents with left flank pain, ASSC in an HbSC adult presenting as isolated back pain without any left flank pain has only been reported twice before.^6,7^ The other such patients suffered increased morbidity and mortality. One required emergent splenectomy and, despite postoperative multisystem organ failure, was able to resume normal activities 6 months after initial presentation.^6^ The other patient died after 2 days due to a rapid decline in Hb concentration, hypovolemic shock, and marked tissue hypoxia; interestingly, this patient also self-reported a history of sickle cell trait.^7^ Isolated back pain in ASSC may be a result of an enlarged spleen contacting the left kidney to irritate Gerota’s fascia, resulting in retroperitoneal pain referred to the back.^6^

Because ASSC has been so infrequently reported in adults with HbSC disease, its natural history in this population is poorly defined, and inciting factors for ASSC in adults have not been clearly delineated, though exposure to high altitude prior to the event is often reported.^7^ In this case, it is possible the patient’s indolent pneumonia served as a stressor that lowered her threshold for ASSC. This patient responded well to RBC exchange transfusion therapy, but some adult HbSC patients do require splenectomy.^8^ No indications for surgical splenectomy have been officially outlined for adults with HbSC, though persistent abdominal pain and/or fever not responsive to transfusion in the setting of ASSC, a life-threatening case of ASSC, and recurrent sequestration crises have been proposed.^6^

ASSC is a rare event in adults with HbSC disease, but clinicians must be aware of the risk for this complication in this population of patients. It is also essential for physicians to ensure these patients understand that their diagnosis of HbSC disease is not the same as sickle cell trait. Targeting on peripheral smear and microcytosis are characteristic findings in patients with HbSC disease that are not present in sickle cell trait. Many questions remain unanswered regarding HbSC. Further research is necessary to fully elucidate the pathophysiology behind ASSC, and the role of splenectomy to treat HbSC patients should be better defined.
